# Identifying Neural Drivers with Functional MRI: An Electrophysiological Validation

**DOI:** 10.1371/journal.pbio.0060315

**Published:** 2008-12-23

**Authors:** Olivier David, Isabelle Guillemain, Sandrine Saillet, Sebastien Reyt, Colin Deransart, Christoph Segebarth, Antoine Depaulis

**Affiliations:** 1 INSERM, U836, Grenoble Institut des Neurosciences, Grenoble, France; 2 Université Joseph Fourier, Grenoble, France; Cuban Neuroscience Center, Cuba

## Abstract

Whether functional magnetic resonance imaging (fMRI) allows the identification of neural drivers remains an open question of particular importance to refine physiological and neuropsychological models of the brain, and/or to understand neurophysiopathology. Here, in a rat model of absence epilepsy showing spontaneous spike-and-wave discharges originating from the first somatosensory cortex (S1BF), we performed simultaneous electroencephalographic (EEG) and fMRI measurements, and subsequent intracerebral EEG (iEEG) recordings in regions strongly activated in fMRI (S1BF, thalamus, and striatum). fMRI connectivity was determined from fMRI time series directly and from hidden state variables using a measure of Granger causality and Dynamic Causal Modelling that relates synaptic activity to fMRI. fMRI connectivity was compared to directed functional coupling estimated from iEEG using asymmetry in generalised synchronisation metrics. The neural driver of spike-and-wave discharges was estimated in S1BF from iEEG, and from fMRI only when hemodynamic effects were explicitly removed. Functional connectivity analysis applied directly on fMRI signals failed because hemodynamics varied between regions, rendering temporal precedence irrelevant. This paper provides the first experimental substantiation of the theoretical possibility to improve interregional coupling estimation from hidden neural states of fMRI. As such, it has important implications for future studies on brain connectivity using functional neuroimaging.

## Author Summary

Our understanding of how the brain works relies on the development of neuropsychological models, which describe how brain activity is coordinated among different regions during the execution of a given task. Knowing the directionality of information transfer between connected regions, and in particular distinguishing neural drivers, or the source of forward connections in the brain, from other brain regions, is critical to refine models of the brain. However, whether functional magnetic resonance imaging (fMRI), the most common technique for imaging brain function, allows one to identify neural drivers remains an open question. Here, we used a rat model of absence epilepsy, a form of nonconvulsive epilepsy that occurs during childhood in humans, showing spontaneous spike-and-wave discharges (nonconvulsive seizures) originating from the first somatosensory cortex, to validate several functional connectivity measures derived from fMRI. Standard techniques estimating interactions directly from fMRI data failed because blood flow dynamics varied between regions. However, we were able to identify the neural driver of spike-and-wave discharges when hemodynamic effects were explicitly removed using appropriate modelling. This study thus provides the first experimental substantiation of the theoretical possibility to improve interregional coupling estimation from hidden neural states of fMRI. As such, it has important implications for future studies on connectivity in the functional neuroimaging literature.

## Abstract 3

Neural long-range interactions can be distinguished from hemodynamic confounds in functional magnetic resonance imaging using new data analysis techniques that will allow experimental validation of models of brain function.

## Introduction

Distinguishing efferent from afferent connections in distributed networks is critical to construct formal theories of brain function [1]. In cognitive neuroscience, the distinction between forward and backward connections is essential in network models [2,3]. This is also important when describing how information is exchanged between different brain systems [4] and how neural coding is embedded in biological networks [5]. Such hierarchical structure is biologically grounded in the asymmetry of connections between neuronal ensembles, as suggested by computational neuroanatomy studies [6–9]. In clinical neuroscience, distinguishing neural drivers (i.e., the source of driving or forward connections in the brain—usually from deep pyramidal cells) from other brain regions is essential when trying to identify structures involved in the origin or in the control of pathological activities. Epileptic seizures are illuminating in that sense. They are characterised by paroxysmal activities which, in the case of focal seizures, originate from the “epileptic focus”, i.e., a neural network restricted to a particular cortical structure, and eventually spread to other structures of the brain [10]. The epileptic focus can thus be interpreted as a neural driver of the pathological activity.

In relation to the existence of distributed networks, theories of brain function have recently promoted the concept of functional integration [11]. Functional integration specifies that brain functions are mediated by transient changes of interactions between certain brain regions, instantiated either by autonomous mechanisms (dynamical systems operating at the limit of stability) or by the action of neural drivers reinforced by the experimental context. In integrated neuroscience, these formal ideas have initiated a search for neural networks using sophisticated signal analysis techniques to estimate the connectivity between distant regions [4,12–18]. At the brain level, connectivity analyses were initiated in electrophysiology (electroencephalography [EEG] and magnetoencephalography [MEG]) because electrical brain signals have an excellent temporal resolution that makes them particularly amenable to such analyses. Connectivity measures in EEG and MEG [13,16] rely on the estimation of metrics of interaction that are more or less related to the notion of temporal precedence (because of propagation and synaptic delays) of the activity in the driving structure with respect to that in the driven ones.

Despite their attractive neurodynamical features, EEG and MEG studies in healthy subjects are limited by their poor spatial resolution. Functional magnetic resonance imaging (fMRI), in contrast, exhibits excellent spatial resolution and has become the method of choice for mapping brain functions. During neuronal activation, fMRI is sensitive mainly to changes of local perfusion and oxygen uptake by neurones [19]. FMRI therefore provides an indirect measure of neuronal activity. The dynamical properties of the technique highly depend on the neurovascular coupling that relates vascular changes to neural activity [20–22]. However, this physiological limitation, which compromises the temporal resolution (∼2 s) of metabolic neuroimaging techniques, has not prevented careful analyses of connectivity using fMRI. Connectivity measurements with fMRI quantify either *functional* connectivity, i.e., the correlation of fMRI time series between different regions [23–25], or *effective* connectivity, i.e., coupling parameters in generative models of fMRI time series [14,15,26]. Although numerous fMRI studies have shown exciting results about brain connectivity, it remains uncertain whether fMRI can be used to identify neural drivers. This is what we propose to evaluate here, in a genetic animal model of absence epilepsy using intracerebral EEG and simultaneous EEG/fMRI recordings.

We use the Genetic Absence Epilepsy Rats from Strasbourg (GAERS) [27]. This animal model has been validated in terms of isomorphism, homology, and pharmacological predictability to be reminiscent of typical absence epilepsy, a form of generalised nonconvulsive epilepsy occurring during childhood in humans [28]. GAERS result from genetic selection of Wistar rats over 80 generations. Animals show spontaneous spike-and-wave discharges (SWDs) associated with behavioural arrest and slight perioral automatisms. These nonconvulsive seizures last 20 s on average and are repeated every minute when the rat is at rest. Intracerebral EEG recordings have shown that the frontoparietal cortex and ventrolateral nuclei of the thalamus play an important role in the generation and/or maintenance of these seizures [27,29]. Using local field potential and intracellular recordings, we have shown recently that SWDs originate from the perioral region of the first somatosensory cortex [30]. A similar finding had earlier been obtained in another genetic model of absence epilepsy [31,32] .

We assess in this study whether fMRI can show evidence of the first somatosensory cortex being a neuronal driver during SWDs. We provide a comparative evaluation of vector regression models (Granger causality) [33] and Dynamic Causal Modelling (DCM) [14]. A key distinction between these models is that Granger causality tests for statistical dependencies among observed (time-lagged) physiological responses, irrespective of how they are caused. In contrast, dynamic causal models represent hidden states that cause the observed data and are therefore causal models in a true sense. If the mapping between the hidden brain states and observed responses is not causal, Granger causality estimated directly from fMRI time series can be very misleading. An example of a noncausal mapping is regional variations in the hemodynamic response function (HRF) that delay hemodynamic responses in fMRI, relative to their hidden neuronal causes (see Protocol S1 for further explanation). Minimising the blurring effects of hemodynamic variability using explicit [34] or implicit (such as in DCM [14]) deconvolution techniques is thus the key aspect of any functional connectivity analysis using fMRI. This paper provides the first, to our knowledge, experimental substantiation of the theoretical possibility to estimate, in fMRI, functional connectivity from hidden neural variables and therefore demonstrates the raison d'être for DCM and other deconvolution techniques.

## Results

Our data analysis involved three distinct components. First, we characterised the hemodynamic response to seizure activity using conventional statistical parametric mapping to identify regionally specific responses. To motivate subsequent analyses of coupling, we then characterised the regional variations in the hemodynamic responses by optimising the parameters of a hemodynamic model for different regions of interest (ROIs) separately. The second component of our analyses comprised a comparative evaluation of Granger causality, before and after deconvolution of hemodynamics, and DCM using key regions identified by the whole brain analyses above. We assessed the significance of directed functional connectivity estimated from the Granger causality measure using surrogate data that removed local time dependencies between regions. To address the equivalent issue with DCM, we used Bayesian model comparison. This entailed comparing a set of models with different directed connections and identifying the model with the largest evidence. The third set of analyses provided an experimental validation of the model selection by analysing directed coupling using intracerebral EEG (iEEG) from the same regions. We used two complementary approaches for cross-validation. First, a simple characterisation of propagation delays, using event-related responses (time-locked to SWDs), enabled us to examine the latency of propagation on a millisecond by millisecond level and establish the direction of connections through temporal precedence. Second, in a series of more elaborate analyses, we used asymmetries in directed generalised synchrony. Using these invasive electrophysiological data, we were able to identify a network model that served as a reference to validate fMRI connectivity analyses.

### EEG Preprocessing

**Figure 1:**
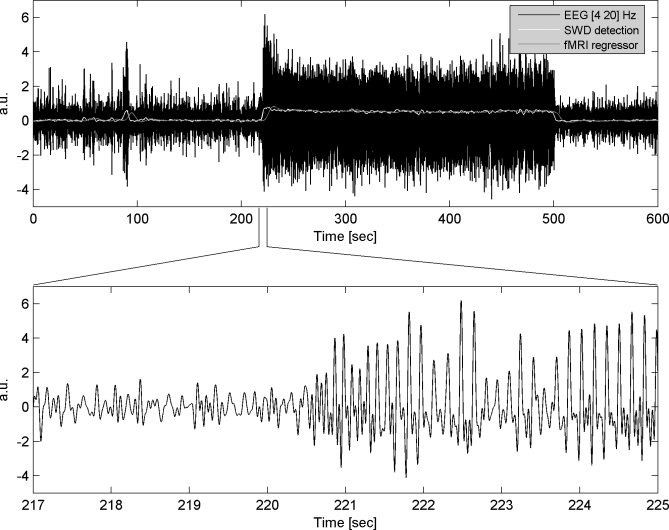
EEG Preprocessing Upper panel: black (“EEG [4 20 Hz]”): 15 min of EEG recordings (band-pass filtered between 4 and 20 Hz) during EPI acquisition obtained in one rat. White (“SWD detection”): EEG power in the 4–20 Hz range (shifted to zero in between SWDs). Grey (“fMRI regressor”): previous EEG power convolved with a canonical HRF. Lower panel: short time window showing the EEG at seizure onset.

EEG recorded during fMRI was of sufficient quality to easily visualise periods of SWDs (Figure 1). Quantification of SWDs was performed by extracting EEG power between 4 and 20 Hz. On average, SWDs showed an increase of power by a factor 2.34 as compared to interictal activity, which corresponded to 2.57 times the standard deviation of interictal power. FMRI regressors were obtained by convolving such EEG power with a canonical HRF [34]. Note that this convolution smoothes and introduces a delay in the SWD time series on the order of several seconds (corresponding approximately to the time to peak of the HRF). FMRI regressors were used to construct statistical parametric maps (SPMs) of regional effects in cerebral blood volume (CBV) related to the occurrence of SWDs.

### Networks Activated during SWDs

**Figure 2:**
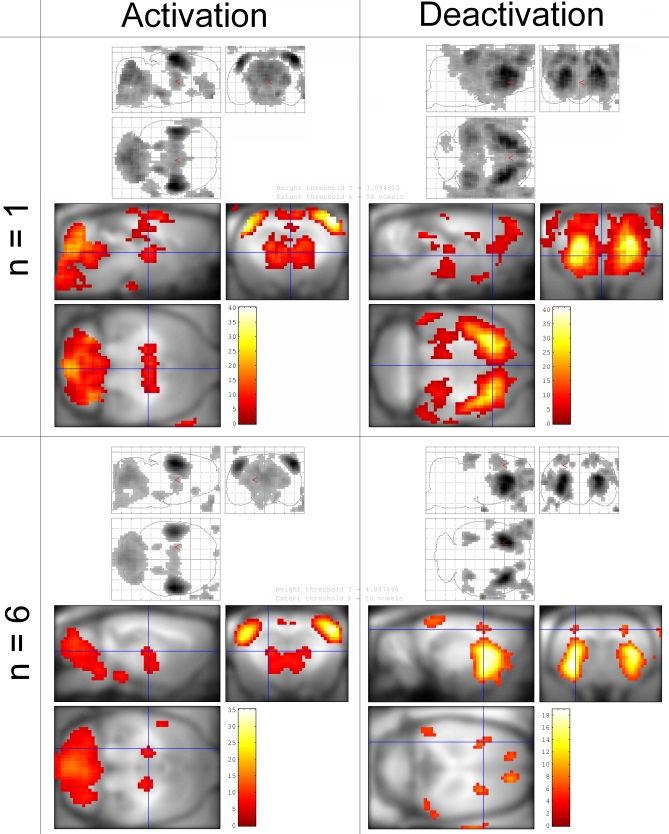
Maps of SWD-Related Changes in CBV Left: activatio*n* = increase of CBV; right: deactivatio*n* = decrease of CBV. Top: typical example of activation/deactivation pattern obtained for a single animal (*n* = 1, *p* < 0.001, FWE corrected). Bottom: activation/deactivation pattern of the group of animals (*n* = 6, fixed effect analysis, *p* < 0.05, FWE corrected). Structures activated at the group level are listed in Table 1.

Highly significant and reproducible seizure-related activations (CBV increases) and deactivations (CBV decreases) were found at the animal level (*p* < 0.001, Familywise Error [FWE] corrected) and at the group level (*n* = 6, *p* < 0.05, FWE corrected) (Figure 2 and Table 1). At the group level, activations were found in the barrel field of the primary somatosensory cortex (S1BF), the centromedial, mediodorsal, and ventrolateral parts of the thalamus (CM/MDL/MDC/CL/PC/VL/Po), the retrosplenial cortex (RSA/RSGb), and the reticular part of the substantia nigra (SNR). These structures are known to be involved in the generation or control of absence seizures. The cerebellum and nuclei of the pons (Mo5) and of the medulla oblongata (MdV) were also found activated. In addition, several areas were found deactivated, such as the striatum (CPu), the limb representation of the primary somatosensory cortex (S1HL/S1FL), the visual cortex (V1M/V1B/V2L), and the secondary motor cortex (M2).

**Table 1:**
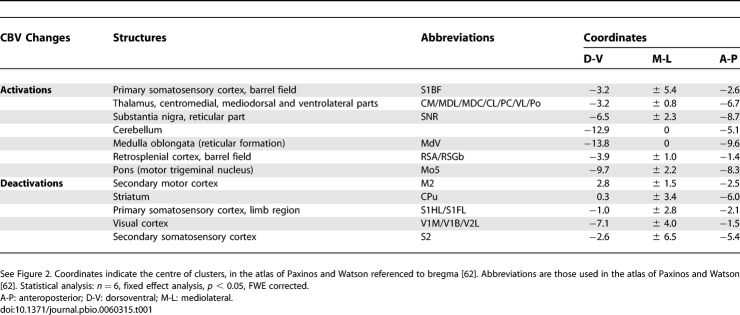
List of Activated and Deactivated Structures during SWDs

### Hemodynamic Response Functions

**Figure 3:**
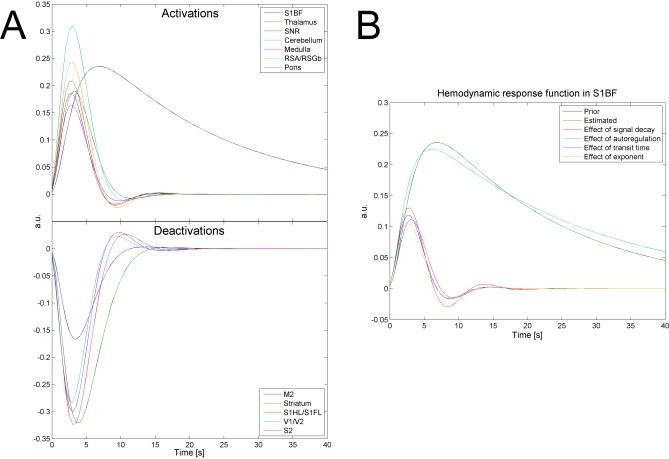
Hemodynamic Response Functions of (De)activated Structures during SWDs (A) Hemodynamics of activated and deactivated structures during SWDs. HRFs for the different ROIs were generated using the median value (see Table 2) of parameters of a truncated hemodynamic model (see Equation 1) adjusted to the ROI time series. (B) From prior values (in blue) of hemodynamic parameters (see Table 2), the effect of each parameter to explain the behaviour of the HRF in S1BF (in green) was evaluated by changing the parameters to their value estimated in S1BF, one at a time. The abnormally slow hemodynamics in S1BF is primarily explained by the strong decrease in the autoregulation constant of the CBF on the vasodilatation (in cyan).

The HRF was found to last significantly longer in S1BF than in other ROIs (Figure 3A). A similar effect was observed in the striatum, to a much lesser extent. These HRFs are kernels of a hemodynamic model, the parameters of which were estimated for every fMRI session. The estimated distribution of hemodynamic parameters in S1BF was found to be significantly different from one of the other ROIs in almost all possible pairs tested (Wilcoxon test, *p* < 0.01 uncorrected; see Table 2). To determine which parameter underlies predominantly the slowness of the HRF in S1BF, we generated different HRFs using prior values of the hemodynamic parameters, with the exception of one parameter, which was set to the value estimated in S1BF (Figure 3B). This allowed us to conclude that the strong decrease of the autoregulation constant γ, instantiating a stable feedback of changes in cerebral blood flow (CBF) on vasodilatatory effects (see Equation 1), is the main cause of the pathological hemodynamics observed.

**Table 2:**
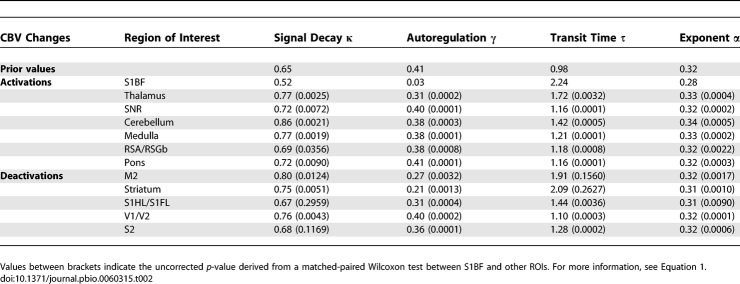
Hemodynamic Parameters Corresponding to the HRF Time Series Shown in Figure 3

These results show a large heterogeneity of HRF waveforms, in particular in S1BF and in the striatum, which has a significant impact on the estimation of connectivity. Estimation of temporal precedence, or of information transfer, and prediction between time series will be affected much by the variability in time to peak of the HRFs. Therefore, these results call for cautious interpretation of causality results directly obtained from hemodynamic measures (see Protocol S1 for a conceptual schematic).

### fMRI Connectivity

#### Granger causality analyses.

**Figure 4:**
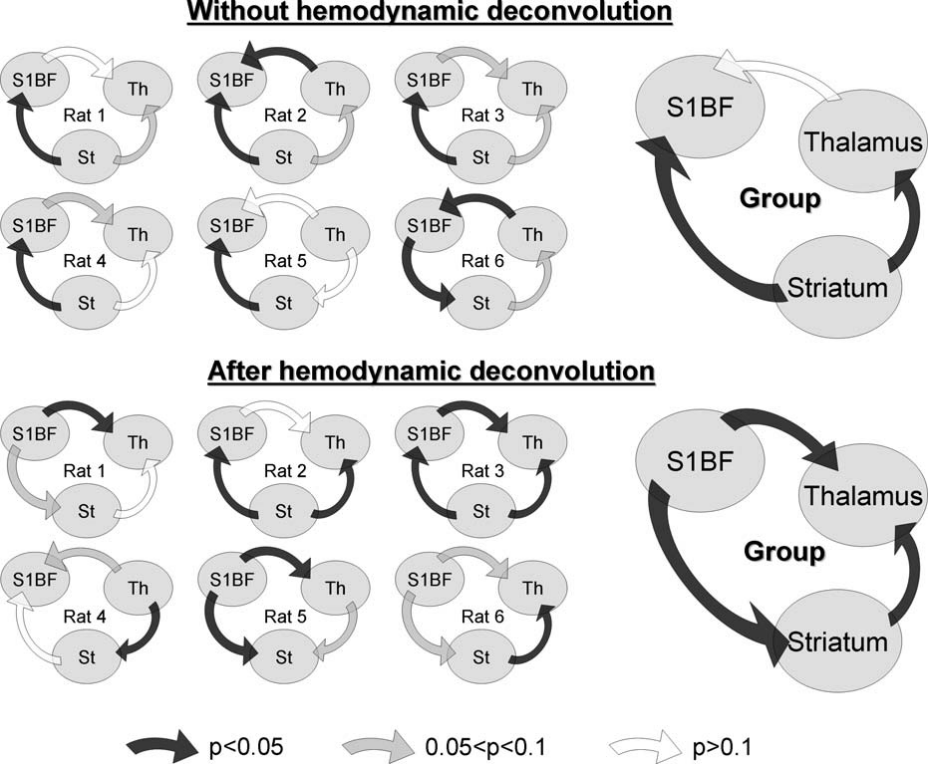
Functional Connectivity Estimated from Granger Causality Oriented networks estimated using the linear measure of Granger causality for each animal (left) and for the group (right), without (top) and after (bottom) hemodynamic deconvolution. For each pair of regions (*X*, *Y*), the directionality and colour of the arrows indicate the sign and statistical significance (obtained from surrogates) of *F_x→y_* − *F_y→x_* (see Equation 4), respectively. See main text for details. St, striatum; Th, thalamus.

The oriented networks estimated using the linear measure of Granger causality applied to CBV-weighted signals directly and to state variables obtained after devonvolution of hemodynamics are shown in Figure 4. At the animal level, significant direction of information transfer is not detected for each connection, and a certain degree of variability is observed between animals. Results at the group level are more significant and easier to interpret. They show a clear distinction between the networks that are estimated without and after deconvolution of hemodynamics. Indeed, direct analysis of fMRI time series leads to the estimation of the striatum as being the driver of the network: significant (*p* < 0.05) driving effects were found from the striatum onto the S1BF (*F_Striatum→S_*_1*BF*_ − *F_S_*_1*BF→Striatum*_ = 0.047, *p* < 0.001) and onto the thalamus (*F_Striatum→Thalamus_*—*F_Thalamus→Striatum_* = 0.011, *p* = 0.023). The interaction between S1BF and thalamus did not show any consistent direction of information transfer (*F_Thalamus→S_*_1*BF*_ − *F_S_*_1*BF→Thalamus*_ = 0.000, *p* < 0.471). In contrast, after deconvolution of hemodynamics, the Granger causality estimated from hidden neural states concludes that S1BF is the neural driver: *F_S_*_1*BF→Striatum*_ − *F_Striatum→S_*_1*BF*_ = 0.017, *p* = 0.038; *F_S_*_1*BF→Thalamus*_ *—F_Thalamus→S_*_1*BF*_ = 0.032, *p* = 0.002; and *F_ Striatum→Thalamus_* − *F_Thalamus→Striatum_* = 0.010, *p* = 0.046.

To sum up, Granger causality at the group level disclosed the predicted architecture in which S1BF drove the other regions, only when applied to hidden neural states. This result clearly demonstrates the important confounding role of hemodynamic variability in functional networks estimated directly from fMRI time series.

#### Dynamic Causal Modelling.

**Figure 5:**
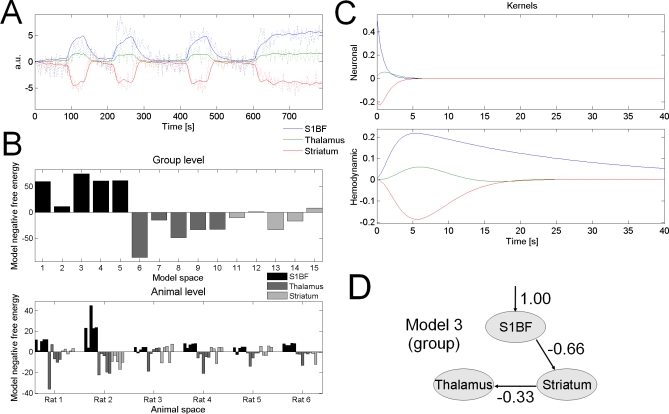
Dynamic Causal Modelling (A) Example showing how DCM (model 1) fitted measured data from a session containing four seizures. (B) Model comparison using the negative free energy (for clarity, the average over the models of the negative energy has been removed). Top: at the group level, the models 1–5 assuming S1BF as being a driver are the most plausible (model 3 is the most plausible at the group level, mainly because of the high value of its evidence in rat 2). Bottom: this result at the group level was found in all rats when pooling over each class. However, in rats 3 and 5, a model assuming the striatum as a driver was found the most plausible (in rat 5, this finding was not significant, i.e., difference of negative energy with a model assuming S1BF as being a driver was lower than three). (C) Neuronal and hemodynamic kernels at the group level obtained from median value of model parameters estimated at the individual level for the most plausible model (model 3, see [B]). (D) Extrinsic connectivity, obtained after averaging matrices *A* and *C* over the animals, for the most plausible model (model 3, see [B]).

Connectivity estimated at the neuronal level (with a conjoint deconvolution of the hemodynamic effects) by DCM revealed the driving role of the first somatosensory cortex S1BF, as may be concluded by comparing the model evidences, at the group level, of the different classes of models tested (Figure 5B, top). This finding was remarkably consistent between animals (Figure 5B, bottom; in Rat 3, however, the most likely model indicated the striatum as the neural driver, but this finding did not survive averaging over model classes). For the most likely model (S1BF driver, model 3, see Figure 5B), Figure 5C shows neuronal and hemodynamic kernels estimated at the group level for each region. Kernels were obtained using the median value of the distribution of model parameters estimated for each session [14]. In agreement with the architecture of the model, neuronal responses of S1BF (in blue) preceded those of the striatum (in red) and of the thalamus (in green). The delay between S1BF and the other regions at half the magnitude of neuronal kernels was about 1.5 s. This value corresponds to the delay observed in intracerebral EEG between first EEG changes in S1BF and the ensuing spread of SWDs to other regions [30]. Interestingly, DCM was able to estimate HRF heterogeneity among regions interconnected at the neuronal level, indicating an effective correction of hemodynamic variability. The HRF in S1BF (in blue) was much slower (half-width = 21 s, κ = 0.97, γ = 0.04, τ = 2.70, and α = 0.32) than that of other regions (thalamus, in green: half-width = 7 s, κ = 0.36, γ = 0.12, τ = 1.75, and α = 0.27; and striatum, in red: half-width = 8.5 s, κ = 0.50, γ = 0.09, τ = 1.99, and α = 0.29), despite the fact that neuronal responses were the fastest in this region. Note that HRFs estimated by DCM were very similar to those estimated without taking into account neuronal connections between regions (Figure 3). Finally, Figure 5D shows extrinsic connectivity, obtained from the median value of the distribution in matrices *A* and *C* (see Equation 5) over animals and sessions, for the most plausible model (model 3, see Figure 5B). Input connectivity strength, decreasing between S1BF (1.00), striatum (0.66), and thalamus (0.33), reflects amplitude of hemodynamic signals recorded (see group *t-*values in Figure 2 and time series in Figure 5A and 5C).

### IEEG Connectivity

#### Spike averaging.

**Figure 6:**
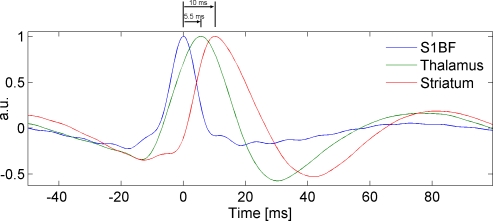
Spike and Wave Complex Averaged over Seizures and Rats The spike observed in S1BF precedes by 5.5 ms and by 10 ms (time to peak) those measured in the thalamus and in the striatum, respectively.

Analysis of the averaged spike-and-wave complex (Figure 6) indicates that the peak of the first spike in S1BF preceded by 5.5 ms and 10 ms those measured in the thalamus and the striatum, respectively. This average sequence of activation was found in all five rats except one in which the spike in thalamus was found to precede the one in S1BF by 2.2 ms. In addition, the average spike recorded in S1BF was sharper and did not show a large slow wave as is the case in the thalamus and in the striatum. These characteristics indicate a specific electrical signature in S1BF, potentially related to its role as neural driver.

#### Generalised synchronisation.

**Figure 7:**
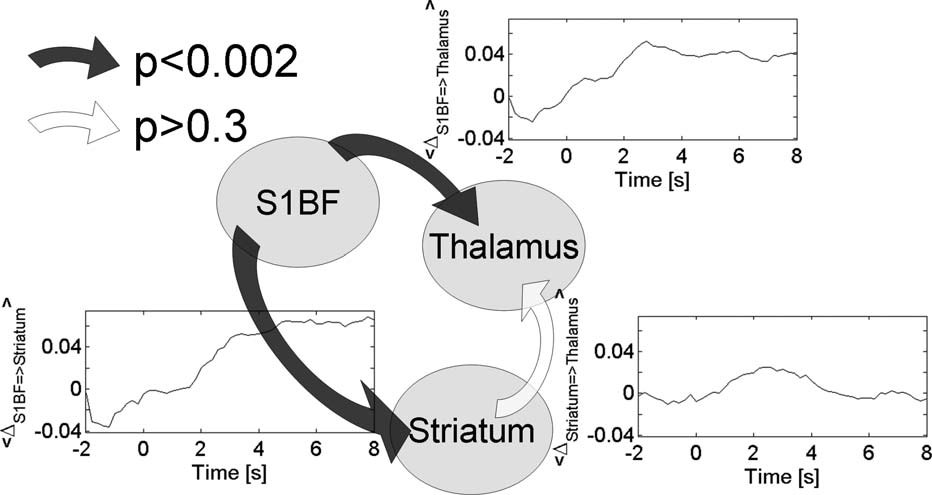
Direction of Information Transfer Estimated in iEEG from the Measure of Generalised Synchronisation A significant and stable, among the first seconds of SWDs, driving effect was found from S1BF towards thalamus and striatum. No consistent directionality was found for the connection between striatum and thalamus.

The oriented network estimated by a measure of generalised synchronisation between iEEG signals was obtained by averaging, for each pair of regions (*X*, *Y*), the interaction measure Δ(*Y* | *X*) − Δ(*X* | *Y*) (see Protocol S2) over seizures and animals between 2 and 8 s after seizure onset (Figure 7). Significant driving effects were found from S1BF onto the striatum (*p* < 10^−9^, Wilcoxon test uncorrected for multiple comparisons) and onto the thalamus (*p* < 0.002). The interaction between striatum and thalamus did not show any consistent direction of information transfer (*p* > 0.39). Connectivity analysis of iEEG signals thus confirmed the role of S1BF as neural driver for thalamic and striatal activity.

## Discussion

In this study, we used a well-recognised animal model of absence epilepsy (GAERS) [27,28] to assess whether fMRI can be used to determine directionality of interactions between remote brain regions. In epilepsy research, estimating neuronal drivers (i.e., epileptogenic zone) within epileptic networks is one of the major issues. In drug-resistant patients with focal epilepsy, for instance, the precise determination of neuronal drivers should have a major surgical impact [35]. This is also true in cognitive neuroscience, in which the possibility to estimate oriented interregional connectivity should permit the refinement of network theories of brain function [3].

Although it is well established that SWDs in absence epilepsy result from paroxysmal oscillations within corticothalamic networks, the respective contributions of the neocortex and of the thalamic relay nuclei in the initiation of such activity are still debated [31,36]. It was first suggested that SWDs originate from a subcortical pacemaker with widespread and diffuse cortical projections [37–41] or from an interaction between cortical and thalamic neurons. However, data from a pharmacological model of SWDs in the cat [42–44] and from a genetic model of absence epilepsy, the Wistar Albino Glaxo/Rijswijk (WAG/Rij) rat [31,32,45], provided evidence for a leading role of the cerebral cortex. In the GAERS, it was found that SWDs are initiated in the facial region of the somatosensory cortex before propagating, or not (for brief SWDs), to the ventrolateral thalamus and to the primary motor cortex [30]. In addition, inhibition of this part of the first somatosensory cortex by local application of tetrodotoxin was shown recently to suppress SWDs (P. O. Pollack, S. Mahon, M. Chavez, and S. Charpier, unpublished data). In human patients with absence epilepsy, fMRI [46] and positron emission tomography (PET) [47] studies showed the involvement of the thalamocortical system during SWDs, but without any clear evidence for the site of initiation of such activity.

Here, using concurrent fMRI and EEG measurements, we obtained SWD-correlated changes in CBV beyond the thalamus and S1. Significant activations or deactivations were also found in the brainstem, cerebellum, SNR, striatum, and different cortices (retrosplenial, visual, limb region of S1, and motor and sensory secondary). Interestingly, all these structures were activated bilaterally, resulting in a symmetrical network. Whereas, to our knowledge, the role of the cerebellum in the generation or control of SWDs has hitherto not been addressed, the spreading of discharges to different cortices was described [48]. CBV changes in striatum and substantia nigra pars reticulata are particularly noteworthy, as these structures, respectively the input and output of the basal ganglia, were suggested to control epileptic seizures in different animal models [49]. For instance, activation of dopaminergic transmission in the striatum suppresses seizures, whereas its inhibition by dopaminergic antagonists aggravates SWDs [50]. Similarly, inhibition of the substantia nigra pars reticulata by pharmacological manipulation is well known to block epileptic seizures in different models, including the GAERS [51]. Our EEG/fMRI results are thus in line with the view that SWDs propagate to different cortical regions, and to subcortical regions as well. Activation of basal ganglia circuits would allow endogenous regulation of SWDs, which can be artificially enhanced by neuromodulation techniques [49].

Two EEG/fMRI studies were performed in the WAG/Rij rat [52,53]. Bilateral activations were also found in the frontoparietal cortex, the thalamus, and brainstem nuclei. No deactivations were reported, however. GAERS and WAG/Rij rats, though similar in many aspects, show also some differences, in particular in the features of spontaneous SWDs [28]. These differences may explain why fMRI activations only partly overlap. Importantly, a strong activation in S1BF is observed in both models. This finding supports the important role of this part of the cortex in the initiation of SWDs, as demonstrated by electrophysiology in GAERS and in Wag/Rij rats [30–32].

In the present study, in addition to revealing the spatial organisation of the epileptic network, we estimated the HRF to SWDs in the different regions involved. We thereby used a truncated hemodynamic model [20] characterised by various parameters directly related to underlying biophysical processes. In the model used, it is assumed that changes in synaptic activity trigger vasodilatatory effects described by the lumped time constant called “signal decay κ.” Vasodilatation induces changes in cerebral blood flow (CBF), which in return have an autoregulation effect on changes in vasodilatation (constant γ in the model). Changes in CBV are then obtained from changes in CBF using a state equation with two parameters (a transit time τ and an exponent α for nonlinear effects). Our main finding here was an abnormally slow HRF in S1BF, due to near suppression of the autoregulation mechanisms of CBF on vasodilatation. The autoregulation constant γ is a lumped parameter that summarises, in dynamical terms, the effects of many different physiological processes involved in the feedback autoregulatory mechanisms occurring during functional hyperemia. Functional hyperemia, which matches the delivery of blood flow to the activity level of each brain region, requires coordinated cellular events that involve neurons, astrocytes, and vascular cells [54]. Deregulation of the function of any of these cell types in S1BF thus appears as a plausible physiological mechanism to explain the abnormally long time constant of CBF feedback that we found. Additional experiments in the future are needed to reveal which processes involved in regulation of vasodilatation by blood flow are exactly altered in the first somatosensory cortex of the GAERS.

Such differences in hemodynamic properties allowed us to challenge the face validity of functional connectivity analyses in fMRI. For simplicity and reproducibility among animals of this validation study of functional connectivity in fMRI, we selected three regions of interest that (1) were the most consistently activated over sessions and animals, (2) exhibited different hemodynamics, and (3) were easily integrated in our current understanding of SWDs. We selected first S1BF because of recent evidence indicating its role as a cortical driver, second the ventrobasal thalamus because it is known that the thalamocortical loop is implicated in SWDs, and third the striatum because of various studies suggesting its role in the control of SWDs. Other structures also showing significant CBV changes at the group level were ignored, either because the signal-to-noise ratio was too low at the session level (because estimated connectivity is related to effect size and highly depends on signal-to-noise ratio, this would have entailed a significant loss of results reproducibility between animals and sessions), or because no experimental evidence was available for validating connectivity results (for instance, it would have been difficult to interpret fMRI connectivity results for cerebellum that has never been explored in GAERS).

The Granger causality measure tested [25,33], heavily based on the concepts of temporal precedence, information transfer, and prediction between time series, estimated the striatum as being the neural driver of SWDs when applied directly to fMRI signals. This result strongly contradicts the evidence from the literature [49]. We then evaluated whether the very same Granger causality measure, but applied to hidden neural states estimated after deconvolution of hemodynamic effects in fMRI time series, would be more compelling. It was indeed the case since S1BF was identified as the neural driver at the group level. Comparison of the results of both analyses demonstrates that the failure of connectivity analysis from original fMRI time series to identify S1BF as the neural driver is due to regional variability of the HRFs. Finally, connectivity analyses at the neuronal level using DCM were also able to reconstruct a meaningful connectivity pattern. Bayesian model comparison showed a clear preference for the models specifying S1BF as the neuronal driver, with consistent reproducibility among animals. At the animal level, results obtained with DCM were more reproducible than with the linear implementation of Granger causality. It is probable that more sophisticated approaches, including multivariate, nonlinear, parametric, or nonparametric implementation of Granger causality [55–57], would have allowed a significant improvement in result reproducibility between animals.

fMRI connectivity analyses were validated using iEEG data obtained in freely moving rats. The directionality of interactions, estimated from the asymmetry of a measure of generalised synchronisation, clearly indicated S1BF as being the driver. The generalised synchronisation measure relies on time-embedding of iEEG signals (Takens' theorem). This manipulation depends upon some parameters that are sometimes difficult to optimise [58], and moreover, its theoretical underpinnings [59] might not be totally fulfilled by brain signals. In view of these potential difficulties, for construct validation in terms of spike propagation, the averaged SWD complex was computed, and a temporal precedence of the activity in S1BF was demonstrated, as anticipated from iEEG generalised synchronisation and from fMRI connectivity.

Because fMRI does not provide sufficient information to reconstruct accurate electrical activity, the neuronal model used in DCM remains necessarily simple, allowing the generation of caricatures of neural states. Nevertheless, DCM distinguished different functional hypotheses in a meaningful way. To our knowledge, this study provides the first experimental validation of DCM for fMRI using invasive EEG recordings. The so-called “synaptic activity” estimated by DCM remains difficult to interpret. First-order electrical kernels (see Figure 5) do not allow the generation of EEG-like signals if convoluted with a random input (as classically done when modelling EEG with neural mass models [60]) because their time constant (∼2 s) is too large to generate the 7–9-Hz oscillations that characterise SWDs in GAERS. Their dynamic properties are more compatible with the rate of change of EEG power often observed at the beginning of seizures (see Figure 1 in [30]). The coupling parameters of DCM might then be interpreted as indications of how changes in EEG power are transferred between regions. Because DCM parameters in fMRI are estimated from several minutes of recordings, the significant difference that was found between models implies that the information transfer is more or less stable during seizures—in other words, that one direction of information transfer dominates. This is indeed what we observed in iEEG, as far as connectivity from S1BF was concerned (Figure 7). Finally, it is important to note that, like any model-based approach, results depend on the assumptions of the generative model used. In particular, current implementation of DCM [14] does not take into account time lags between neural populations due to conduction velocities and propagation through dendritic trees. Elaborating and validating a more realistic neural model for DCM in fMRI taking time dependencies into consideration would be interesting, but goes well beyond the scope of this work.

This study is, to our knowledge, the first electrophysiological validation of fMRI connectivity analyses based on Granger causality and Dynamic Causal Modelling using a well-characterised animal model of functional coupling. As such, it has important implications for such studies that are starting to predominate in the functional neuroimaging literature on connectivity. Our results clearly indicate that one must minimise spurious interactions due to hemodynamic variability between brain regions using explicit or implicit (such as in DCM) deconvolution of hemodynamic effects in fMRI time series. Otherwise, directed functional connectivity results should be taken cautiously, particularly if one cannot demonstrate that hemodynamic properties are the same in every region analysed.

## Materials and Methods

### Animal preparation and data acquisition.

Experimental procedures and animal care were carried out in accordance with the European Community Council Directive of 24 November 1986 (86/609/EEC). They were approved by the Ethical Committee in charge of animal experimentation at the Université Joseph Fourier, Grenoble (protocol number 88–06). Six male adult GAERS (281 ± 56 g) were used for the fMRI/EEG study, and five adult GAERS (two males, three females; 232 ± 70 g) were recorded in iEEG.

### fMRI/EEG experiments.

Spontaneous seizures were measured during magnetic resonance (MR) experiments using EEG. Animals were equipped with three carbon electrodes located on the skull near the midline (frontal, parietal, and occipital), several hours prior to the MR experiments. Two additional carbon electrodes were used to monitor cardiac activity (electrocardiography [ECG]). Because absence epilepsy is suppressed by anaesthesia, animals were maintained conscious under neuroleptanalgesia.

Anaesthesia was induced under 5% isoflurane, maintained under 2% isoflurane during animal preparation, and stopped during MR acquisition. The femoral artery was catheterised to allow administration of an iron-based superparamagnetic contrast agent (injected as a bolus just before MR preparatory settings, 8 mg Fe/kg, i.e., 145 mmol Fe/kg, Sinerem) and infusion of curare and analgesics. Just before inducing neuroleptanalgesia, a tracheotomy was performed, and animals were ventilated at 90 breaths/min throughout the rest of the experiment. Neuroleptanalgesia was induced using an intravenous bolus of d-tubocurarine (1 ml/kg). Animals were then maintained under intravenous infusion of a mixture of d-tubocurarine (1.2 mg/kg/h), Fentanyl (3 μg/kg/h), and haloperidol (150 μg/kg/h) [30].

Animals were secured in an MR-compatible, customised, stereotaxic headset with ear and tooth bars. They were positioned in the magnet, maintained in position between 3 and 4 h for data acquisition, and then sacrificed. Rectal temperature was monitored and kept at 37 °C using a heating pad positioned under the animal.

MR imaging was performed in a horizontal-bore 2.35 T magnet (Bruker Spectrospin), equipped with actively shielded magnetic field gradient coils (Magnex Scientific) and interfaced to a SMIS console (SMIS). A linear volume coil was used for excitation (internal diameter 79 mm), and a surface coil was used for detection (Rapid Biomedical). Both coils were actively decoupled.

T_1_-weighted anatomical images were acquired using a 3D-MDEFT sequence with parameters optimised following the procedure described in [61]: voxel size = 0.333 × 0.333 × 0.333 mm^3^, TI = 605 ms, quot = 0.45, alpha = 22°, TR/TE = 15/5 ms, and BW = 20 kHz. CBV-weighted measurements were made with gradient-echo echo-planar imaging (EPI) acquisition (two shots, data matrix = 48 × 48, FOV = 35 × 35 mm^2^, 15 contiguous 1.5-mm-thick slices covering the whole brain, alpha = 90°, TE = 20 ms, TR = 3 s). Functional volumes were acquired over about 2 h, in several 30-min sessions to prevent overheating of the gradient hardware. 3D-MDEFT and EPI images were centred to facilitate superimposition.

EEG and ECG signals were sampled simultaneously with fMRI at 1,024 Hz (SD32, Micromed). ECG was merely used to monitor the physiological state of animals. When ECG revealed a heart frequency below 250 beats/min, the experiment was terminated, and the animal was sacrificed. EEG and fMRI temporal coregistration was ensured by the EEG acquisition software recording a TTL signal from the MR system at each volume acquisition.

### iEEG experiments.

For the iEEG recordings, GAERS were implanted with intracerebral electrodes under general anaesthesia (diazepam 4 mg/kg intraperitoneally [i.p.], ketamine 100 mg/kg i.p.). Pairs of electrodes formed of stainless steel wires (0.175 mm) separated by 2 mm on the longitudinal axis were stereotaxically placed in each structure targeted. Stereotactic coordinates were as follows, with the bregma as reference [62]: (1) first somatosensory cortex S1BF (anteroposterior [AP]: −1 and −3 mm; mediolateral [ML]: +5 mm; and dorsoventral [DV]: −3 mm), (2) ventrobasal thalamus (AP: −2.3 and −4.2 mm; ML: +2.4 mm; and DV: −6.2 mm), and (3) striatum (AP: +3 and −0.8 mm; ML: +3 mm; and DV: −6 mm). Two additional electrodes (stainless steel screws) were fixed in the nasal and occipital bones to serve as reference and/or ground. All electrodes were connected to a female microconnector that was fixed to the skull by acrylic cement. Animals were allowed to recover for a week, during which they were handled daily for habituation. Once implanted, the rats were kept alive 2 mo at maximum. They were killed by an overdose of pentobarbital, and their brains were then removed and cut into 20-μm coronal sections. These sections were stained with cresyl violet, and each site was localised with reference to the atlas of Paxinos and Watson [62,63]. Electrode implantation was considered correct if the centre of gravity of the pair of electrodes was located within the targeted structure.

Electroencephalograms were recorded in awake, freely moving animals, using a digital acquisition system (Cambridge Electronic Design) with a sampling rate of 2 kHz and analog filters (high-pass filter 1 Hz/low-pass filter 90 Hz). During the recording sessions, rats were continuously watched to detect abnormal posture or behaviour. Sessions did not exceed 2 h and were performed between 9:00 am and 5:00 pm.

### fMRI/EEG data analysis.

fMRI data analysis was done using SPM5 (Statistical Parametric Mapping, Wellcome Department of Imaging Neuroscience, Functional Imaging Laboratory, London, UK). Some routines of this software were adapted to rat imaging in accordance with [63].

### Spatial preprocessing.

For each session, EPI volumes were first realigned to account for motion correction. All images were then normalised to a 3D-MDEFT template with coordinates chosen according to the rat atlas of Paxinos and Watson, with the origin at the bregma [62]. Normalised images were resampled to reach an isotropic spatial resolution of 0.4 mm. Finally, normalised EPI images were smoothed with a Gaussian kernel of 0.5-mm width. Statistical analysis was done on smoothed, normalised, and realigned EPI images.

### Statistical maps of SWD-related regional CBV changes.

Statistical maps of regional CBV changes in relation to SWDs were obtained using the standard procedure applied in EEG/fMRI studies of epilepsy [64,65]. It consists of the detection of epileptic events in the EEG. A regressor of interest for fMRI data is then obtained by convolving EEG epileptic events with a model of the hemodynamic impulse response function [66]. If the impulse response is causal (which is usually the case), it is assumed that electrical activity precedes and causes hemodynamic changes.

SWDs were extracted from the EEG using a moving average (time window length = 2 s; sampling rate = 5 Hz) of EEG power between 4 Hz and 20 Hz. SWD power was then scaled such as to be about zero between SWDs and about one during SWDs. Note that it was not necessary to correct imaging or cardiac artefacts in our data because they were not significant at frequencies of SWDs. The SWD regressor used for fMRI statistical analysis was obtained by convolving the normalised SWD power with the canonical HRF provided in SPM5.

For each animal, SPMs of the *t*-statistic of SWD-related activations were obtained by correlating the high-pass filtered (cutoff = 0.97 mHz) time series of each voxel with the SWD regressor using a standard first-level multisession statistical design [67]. Activations at the group level were obtained using a fixed-effect analysis following guidelines provided in [68]. The decision to perform a fixed-effect analysis was based on (1) the reduced number of animals (*n* = 6) being too small to perform a random-effect analysis and (2) the excellent reproducibility between animals of the activation patterns.

### Estimation of hemodynamic parameters in regions activated during SWDs.

Activation maps were obtained under the conventional hypothesis of identical hemodynamics all over the brain. Although this assumption is particularly convenient to obtain statistical maps, significant hemodynamic variability is to be expected [69–71]. Taking into account this spatial variability is critical in identifying neuronal drivers from fMRI signals. We therefore estimated the HRFs in the different structures activated.

A biophysical model of brain hemodynamics was used to biologically constrain the estimation of the HRFs. We therefore adapted the hemodynamic model used in [14,20] to the measurement of CBV-weighted signals (due to the use of an iron contrast agent). Briefly, we removed from the distributed version of DCM (SPM5, http://www.fil.ion.ucl.ac.uk/spm/) the state equation corresponding to the definition of deoxyhemoglobin content, and we changed the output equation that was developed for BOLD signals (assuming BOLD signals as arising from a mixture of CBV and blood oxygenation effects). The model thus described below is a truncated version of the hemodynamic model developed in [20]. For the *i*th region, neuronal activity *z_i_* causes an increase in a vasodilatory signal *s_i_* (time constant κ*_i_*) that is subject to autoregulatory feedback (autoregulation constant γ*_i_*). Inflow *f_i_* responds in proportion to this signal with changes in blood volume *v_i_* (time constant τ*_i_* and nonlinear constant α*_i_*):


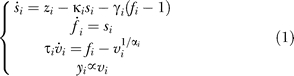


Variations in CBV-weighted signals *y* were assumed to be proportional and of opposite sign to variations of blood volume *v*—a CBV increase shortens the transverse relaxation time [72]. The four hemodynamic parameters for each region *i* (κ*_i_*, γ*_i_*, τ*_i_*, and α*_i_*) were estimated from the time series of each ROI using a maximisation-expectation algorithm [73], similar to the one used in the standard DCM procedure [14].

### Deconvolution of hemodynamics.

Most methods to infer the direction of information transfer between two time series are based on identifying temporal precedence. If past activity of a given region *X* helps predicting current activity of another region *Y*, then it is assumed that the activity of *X* causes to some extent the activity of *Y*. Although compelling, temporal precedence in fMRI time series may be biased by regional variability of hemodynamics (see Protocol S1 for an intuitive explanation). Consequences of hemodynamic variability can be minimised by deconvolving fMRI time series with a hemodynamic impulse response function. Output time series represent then hidden state variables that are more closely related to neuronal activity. Instead of original fMRI time series, such a state-space model can be used to infer functional connectivity.

Hemodynamic deconvolution of each ROI time series was performed as described in [34]. Under linear assumption, fMRI signals *m*(*t*) can be modelled as the result of the convolution of neural states s(*t*) with a hemodynamic response function h(*t*):


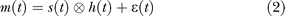


where *t* is the time and ⊗ denotes convolution. *ɛ*(*t*) is the noise in the measurement, assumed here to be white and therefore defined by its constant power spectrum 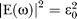 . The estimation 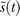 of the neural states *s*(*t*) was obtained using the following formula [34]:


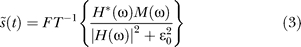


where *FT* ^−1^ denotes the inverse Fourier transform, and *H*(ɛ), *M*(ɛ) are the Fourier transform of *h*(*t*), *m*(*t*), respectively. For each ROI, the hemodynamic response function *h*(*t*) was obtained after optimising the parameters of the biophysical model described in Equation 1. The expectation-maximisation algorithm used for this parameter optimisation also provided the value of the noise power spectrum ɛ_0_^2^.

### Granger causality analysis.

Granger causality measures have been proposed recently to identify the direction of information transfer between remote brain regions recorded in fMRI [25,33]. In its simplest version, Granger causality is computed using linear multivariate autoregressive models of fMRI time series. For each pair of brain regions *X* and *Y*, the linear influence from *X* to *Y* (*F_x→y_*) and from *Y* to *X* (*F_y→x_*) is defined as follows [25]:


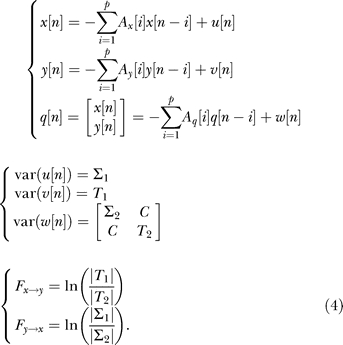


where *x* and *y* are the time series of regions *X* and *Y*. In Results, they correspond either to hemodynamic activity (fMRI signals entered directly into the analysis) or to hidden neural states (obtained from fMRI signals using Equation 3). *x*[*n*] corresponds to the *n*th time bin of *x*. The three first lines of Equation 2 define autoregressive models for time series of regions *X* and *Y*, the three lines below quantify the residual variances, i.e., how well autoregressive models predict time series, and the two last lines show how interdependency measures are defined from the residual variances. Autoregressive models were estimated using the Matlab package ARfit (http://www.gps.caltech.edu/~tapio/arfit/) [74,75]. The model order *p* was defined according to the Schwarz Bayesian criterion [76]. It measures the efficiency of the parameterised model in terms of predicting the data and penalises the complexity of the model, where complexity refers to the number of model parameters.

For each pair of regions (*x*, *y*), statistics on the asymmetry of the interaction measure *F_x→y_* − *F_y→x_* were obtained using 999 surrogate datasets [77] that were constructed for each session by translating, independent of each another, ROI time series by a random number of time samples. Surrogates thus destroyed local time interdependencies and preserved the properties of each signal taken separately. They allowed one to estimate distributions of Granger causality under the null hypothesis that ROI time series were locally uncorrelated and were not time-locked over sessions and animals. Null distributions were drawn at the animal and group levels by averaging *F_x→y_* − *F_y→x_* over sessions and animals for each surrogate realisation. *p*-Values on the direction of interactions were obtained by comparing the value computed from original data to the null distribution constructed from surrogates (see [77] for a review on surrogates).

We refer here to the simplest implementation of Granger causality because it is the most popular in fMRI [25,33]. However, there are many other possibilities, including parametric and nonparametric nonlinear approaches that have been applied to the brain, mainly in electrophysiology [55–57].

### Dynamic Causal Modelling.

DCM [14] relies on a biophysical model that connects the neuronal states *z*, called “synaptic activity,” to fMRI signals. A bilinear neuronal state equation specifies the connectivity between *n* brain regions:


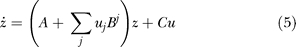


where *A*, *B*, and *C* are connectivity matrices, and *u* are inputs to the neural system. The synaptic activity is then transformed into fMRI signals using the hemodynamic model described in Equation 1. Using a maximisation-expectation algorithm, DCM proceeds to a conjoint estimation, from the measured CBV time series, of the neuronal parameters (connectivity matrices *A*, *B*, and *C*) and of the four hemodynamic parameters for each region *i* (κ*_i_*, γ*_i_*, τ*_i_*, and α*_i_*). In other words, it performs in one step the hemodynamic deconvolution and connectivity estimation between hidden neural variables. This implies a certain degree of interactions between both processes that potentially results in more robust results than when deconvolution and connectivity analyses are taken separately.

**Figure 8:**
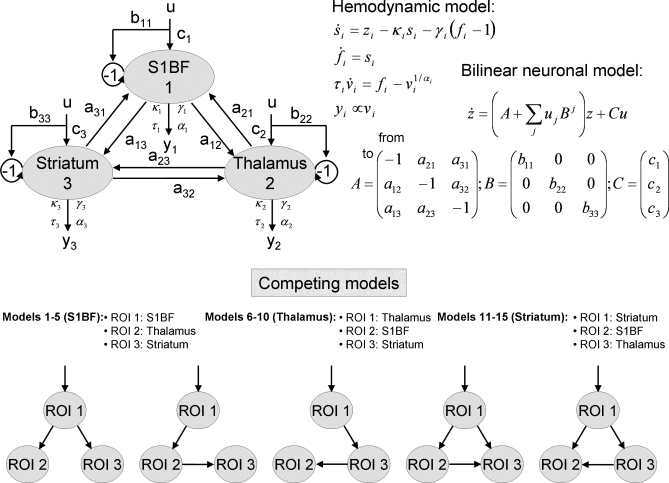
Dynamic Causal Model: Architecture and State Equations Input *u* corresponds to detected epileptic events in the EEG. All parameters of the models are estimated from data *y* (CBV-weighted fMRI signals) using a Bayesian framework. Different configurations of the interregional connectivity A as shown in the competing models are used to estimate the putative neural driver, based on Bayesian model comparison. The 15 possible unidirectional models were generated from the five models shown in this figure using permutations on the ROI names (S1BF driver: models 1–5; thalamus driver: models 6–10; striatum driver: models 11–15). See main text for additional details.

For the present study, we identified neural drivers within a small network composed of three regions. To prevent introducing any bias in the estimation of functional connectivity, we did not take into account prior anatomical information about probable missing connections. We thus chose to specify all possible unidirectional networks comprising direct and/or indirect connections (15 models; S1BF driver: models 1–5; thalamus driver: models 6–10; striatum driver: models 11–15) (Figure 8). Because DCM necessitates knowledge of the inputs *u*, we defined *u* as being equal to the SWD regressor—shifted backwards in time (400 ms, which corresponds approximately to the time constant of the driver's DCM neuronal kernel, see results in Figure 5) to account for neuronal filtering (*u* is a presynaptic input whereas the EEG reflects multi-postsynaptic activity [60]). In each model, an input *u* (non-zero *C* matrix) was applied to the assumed neural driver. Here, input *u* must be thought of as a practical way to model unstable dynamics intrinsically generated by an epileptic focus, using simple dissipative neural models used by DCM as described in Equation 5. In the models shown in Figure 8, the region receiving input *u* transfers the information to other regions with forward connections (matrix *A*). For parsimony, we did not allow a modulation of the interregional connection strength by *u* (by the means of the modulatory matrix *B*). We thereby assumed that the connection strength did not vary between ictal and interictal states. To conform to standard practice in DCM studies, only self modulation (first diagonal of *B*) of the region receiving the exogenous input was allowed. Actually, because it appears that inputs *u* were very close to zero during interictal states, assumptions about connectivity modulation had little effect on the parameters estimated.

Identification of the neural driver in the 15 competing models (Figure 8) was done using Bayesian model comparison based on model evidence [78]. Practically, the model log-evidence was approximated by the model negative free energy, the criterion used for optimising the model parameters [14], which is a tight lower bound on the log-evidence. The most plausible model is the one with the largest negative free energy, i.e., the best fit to the data. A difference in log-evidence of approximately three is usually taken as strong evidence for one model over the other (i.e., the marginal likelihood of one model is ∼20 times the other) [14]. Assuming each dataset is independent of the others, the log-evidence at the group level (or at the animal level when different sessions have been acquired) is simply obtained by adding the log-evidence of each session [79].

### IEEG data analysis.

iEEG data analysis was done using a SPM5 Toolbox for intracerebral EEG developed in our laboratory. iEEG signals were first band-pass filtered between 5 and 100 Hz to capture the main frequencies of SWDs and to remove motion artefacts in low EEG frequencies. Seizures were visually detected. Only those showing (1) no movement artefact, (2) preictal and postictal periods of at least 4 s, and (3) a duration of at least 10 s were kept for further analysis (*n* = 72).

As a first estimation of the sequence of “activation” within the three implanted structures, spike averaging over time was performed. An ad hoc algorithm, based on EEG amplitude thresholding and local maxima identification, was implemented in which the first peak of the SWD complex was detected in signals originating from S1BF. The mean activation pattern was then obtained by averaging each SWD complex over time, seizures and animals using a time window covering from 50 ms before up to 80 ms after detected spikes. The delay between the peaks in the signals from the different structures was finally measured on the averaged waveforms.

Further functional connectivity analyses in iEEG were performed using a nonlinear measure based on the concept of generalised synchronisation [13,16,80,81]. By definition, generalised synchronisation exists between two dynamical systems *X* and *Y* when the state of the response system *Y* is a function of the state of the driving system *X*:*Y* = *F*(*X*). If *F* is continuous, two close points on the attractor of *X* should correspond to two close points on the attractor of *Y*. An important feature of generalised synchronisation is that synchronised time series can look very dissimilar, which is critical for analysing highly nonlinear signals such as those measured with EEG in epilepsy.

Details of these methods can be found in Protocol S2. Briefly here, we used the normalised measure of generalised synchrony Δ between regions X and Y as described elsewhere [13,16]. There are two ways to compute Δ, which we denote Δ(*X* | *Y*) and Δ(*Y* | *X*). Δ(*X* | *Y*) and Δ(*Y* | *X*) are not identical for asymmetrical systems. This property can be used to dissociate the driver and the driven systems, and we defined the direction of information transfer between *X* and *Y* using Δ(*Y* | *X*) − Δ(*X* | *Y*). For each seizure, the normalised measure of generalised synchronisation Δ was computed on a time window (duration of 4 s to get sufficient number of time points for robust estimation of generalised synchronisation), which was translated every 200 ms between −2 s up to 8 s according to seizure onset. By using a sliding window, we were able to compute the evidence for directed connectivity as a function of peristimulus time, after SWDs onset.

## Supporting Information

Protocol S1 Time Precedence and Neuronal Causality in fMRI Time Series Reported is an intuitive view of the blurring effects of hemodynamics for the estimation of directional connectivity.
(41 KB DOC)

Protocol S2 Measures of Generalised Synchronisation Reported are concepts and detailed equations used to quantify generalised synchronisation.
(86 KB DOC)

## Abbreviations

CBF: cerebral blood flow
CBV: cerebral blood volume
DCM: Dynamic Causal Modelling
EEG: electroencephalography
fMRI: functional magnetic resonance imaging
FWE: Familywise Error
HRF: hemodynamic response function
iEEG: intracerebral EEG
MEG: magnetoencephalography
ROI: region of interest
S1BF: barrel field of the primary somatosensory cortex
SWD: spike-and-wave discharge
