# Supplementary material for: Identifying Neural Drivers with Functional MRI: An Electrophysiological Validation
Source: PLoS Biol. 2008 Dec 23;6(12):e315. doi: 10.1371/journal.pbio.0060315 (PMC2605917; doi:10.1371/journal.pbio.0060315)
Supplement: Protocol S1 — Reported is an intuitive view of the blurring effects of hemodynamics for the estimation of directional connectivity. (41 KB DOC) [file pbio.0060315.sd001.doc]

# Time precedence and neuronal causality in fMRI time series

Granger Causality Mapping (GCM) is based on temporal precedence between time series of different regions: if the past activity of a given region X helps predicting the current value of the activity of another region Y, then it is assumed that the activity of X causes to some extent the activity of Y. Although compelling, temporal precedence in fMRI time series is potentially a confounding concept because of the regional variability of hemodynamic properties. Indeed let us assume two regions X and Y which are interconnected with synaptic efficacies stronger from region X to Y. Then at the electrical level, usually a delay is observed between X and Y time series (at least in evoked responses to an event, see Figure). It is generally assumed that the neuronal activity then triggers a cascade of metabolic and hemodynamic events which can be summarised under a hemodynamic filter, the impulse response, which is equivalent to the HRF. If the hemodynamic filter is similar in regions X and Y, then one can hope to estimate the proper orientation of information transfer when using fMRI signals (Figure, left). However, if there is some interregional variability in hemodynamics, and in particular if the HRF is much slower in X than in Y (Figure 1), then the estimated causality at the fMRI level may be wrong. This is an important confound in any causality measure based on hemodynamic signals that was directly addressed in this study.


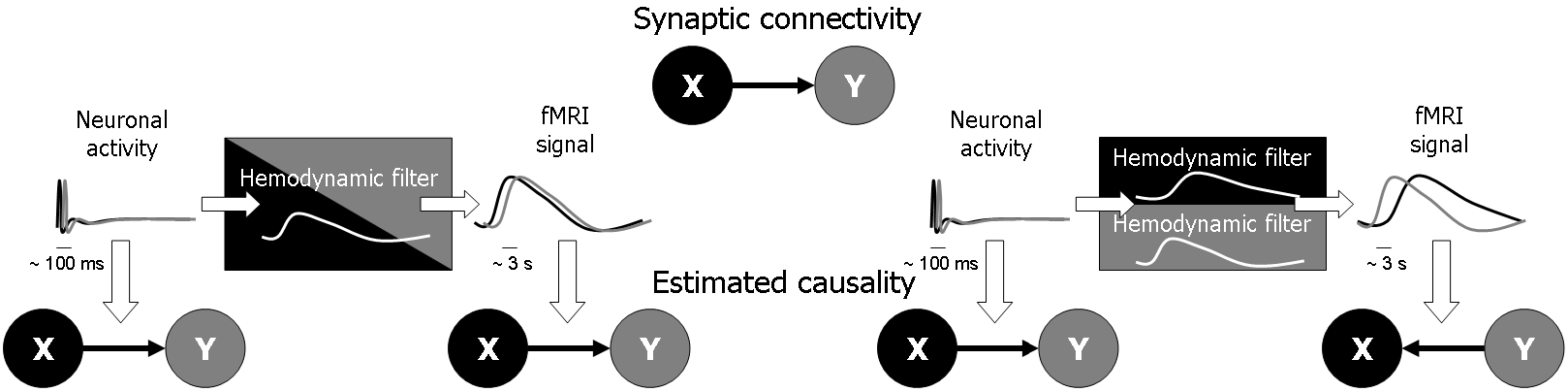


**Figure:** Two regions X (black) and Y (grey) show strongest synaptic connections from X to Y. Such synaptic configuration induces a temporal precedence of X over Y in neural responses to a stimulus. Therefore estimated causality at the neural level usually reflects the orientation of synaptic connectivity. Neuronal activity is then transformed into fMRI signals by the means of a hemodynamic filter. If there is no difference in that filter (left), then the estimated causality is equivalent to the orientation of synaptic connectivity. However, if there is some variability, in particular if hemodynamic processes are slower in X than in Y (right), then the estimated causality at the hemodynamic level may be wrong.
