# Supplementary material for: Identifying Neural Drivers with Functional MRI: An Electrophysiological Validation
Source: PLoS Biol. 2008 Dec 23;6(12):e315. doi: 10.1371/journal.pbio.0060315 (PMC2605917; doi:10.1371/journal.pbio.0060315)
Supplement: Protocol S2 — Reported are concepts and detailed equations used to quantify generalised synchronisation. (86 KB DOC) [file pbio.0060315.sd002.doc]

# Measures of generalised synchronisation

Algorithms that quantify generalised synchronisation all rely upon some time-delay embedding [1]. This method reconstructs the state space using *m*-dimensional vectors whose components are consecutive values of the signals $\vec{X}_{n}=\left[x_{n},x_{n+\tau},...,x_{n+\left(m-1\right)\tau}\right]$, $\vec{Y}_{n}=\left[y_{n},y_{n+\tau},...,y_{n+\left(m-1\right)\tau}\right]$, $n=1,...,N'$, where $N'=N-\left(m-1\right)\tau$, $m$ is the embedding dimension and $\tau$ is the delay time. Here, $m$ was systematically set to 3, according to a geometrical method to determine $m$ [2] that was first run on typical segments of measured time series. The delay time parameter $\tau$ was chosen to be the first zero of the auto-correlation function of the signals. In addition, a Theiler correction equal to $\tau$ was used to diminish spurious correlation [3].

The existence of F induces a particular relationship among the neighbourhood structure of states on the attractors of coupled systems. This structure can be measured in terms of closeness between $\vec{X}_{n}$ and its corresponding actual and mutual neighbours, using the geometric definition of distance [4]. Let and , $j=1,...,k$, denote the time indices of the *k* nearest neighbours of $\vec{X}_{n}$ and $\vec{Y}_{n}$. By definition, are the true neighbours of $\vec{X}_{n}$ and are the mutual neighbours of $\vec{Y}_{n}$, and *vice versa* for . For each $\vec{X}_{n}$, the mean squared Euclidean distance to its *k* nearest neighbours is defined as

(4)

and the *Y*-conditioned mean squared Euclidean distance of $\vec{X}_{n}$ is defined by replacing the true nearest neighbours of $\vec{X}_{n}$ by the equal partners of the closest neighbours of $\vec{Y}_{n}$ (mutual neighbours of $\vec{X}_{n}$):

. (5)

If the systems *X* and *Y* exhibit generalised synchrony, then and thus $D_{n}^{\left(k\right)}\left(X|Y\right)\approx D_{n}^{\left(k\right)}\left(X\right)$. Conversely, if the systems are independent then $D_{n}^{\left(k\right)}\left(X|Y\right)\approx D_{n}^{\left(N'-1\right)}\left(X\right)$ where $D_{n}^{\left(N'-1\right)}\left(X\right)$ is the mean squared Euclidean distance between point $\vec{X}_{n}$ and the remaining points $\vec{X}$. As normalised measure of generalised synchrony, we will use [4,5]:

$\Delta^{\left(k\right)}\left(X|Y\right)=\left\langle\frac{D_{n}^{\left(N'-1\right)}\left(X\right)-D_{n}^{\left(k\right)}\left(X|Y\right)}{D_{n}^{\left(N'-1\right)}\left(X\right)}\right\rangle_{n}$ (6)

which is below, but close to, 1 when *X* and *Y* are synchronised, and tends towards 0 when they are independent.

$\Delta^{\left(k\right)}\left(Y|X\right)$ is obtained in a similar fashion, but is not identical to $\Delta^{\left(k\right)}\left(X|Y\right)$ for asymmetrical systems. This property can be used to estimate the driver and driven systems, and we define the direction of information transfer between X and Y using $\Delta^{\left(k\right)}\left(Y|X\right)-\Delta^{\left(k\right)}\left(X|Y\right)$.

The number of neighbours was chosen as $k=2\times m$. For each seizure, the normalised measure of generalised synchronisation $\Delta$ was computed on a time window (duration of 4 s), which was translated every 200 ms between -2 s up to 8 s according to seizure onset. By using a sliding window, we were able to compute the evidence for directed connectivity as a function of peristimulus time, after SWDs onset (see Results).

# References

1. Takens F (1981) Detecting strange attractors in turbulence. In: Rand DA, Young LS, editors. Berlin: Springer. pp. 366.

2. Kennel MD, Brown R, Abarbanel HDI (1992) Determining embedding dimension for phase-space reconstruction using a geometrical construction. Phys Rev A 45: 3403-3411.

3. Theiler J (1986) Spurious dimension from correlation algorithms applied to limited time-series data. Phys Rev A 34: 2427.

4. Quian Quiroga R, Kraskov A, Kreuz T, Grassberger P (2002) Performance of different synchronization measures in real data: a case study on electroencephalographic signals. Phys Rev E 65: 041903.

5. David O, Cosmelli D, Friston KJ (2004) Evaluation of different measures of functional connectivity using a neural mass model. Neuroimage 21: 659-673.
